# Development of a versatile resource for post-genomic research through consolidating and characterizing 1500 diverse wild and cultivated soybean genomes

**DOI:** 10.1186/s12864-022-08326-w

**Published:** 2022-03-31

**Authors:** Hengyou Zhang, He Jiang, Zhenbin Hu, Qijian Song, Yong-qiang Charles An

**Affiliations:** 1 Donald Danforth Plant Science Center, St Louis, MO 63132 USA; 2 US Department of Agriculture, Agricultural Research Service, Soybean Genomics and Improvement Laboratory, Beltsville, MD 20705 USA; 3 US Department of Agriculture, Agricultural Research Service, Midwest Area, Plant Genetics Research Unit, 975 N Warson Rd, St. Louis, MO 63132 USA

**Keywords:** Soybean, Single nucleotide polymorphism (SNP), Genetic diversity, Whole-genome resequencing, US soybean germplasm collection, Linkage disequilibrium (LD), Genomic equivalence

## Abstract

**Background:**

With advances in next-generation sequencing technologies, an unprecedented amount of soybean accessions has been sequenced by many individual studies and made available as raw sequencing reads for post-genomic research.

**Results:**

To develop a consolidated and user-friendly genomic resource for post-genomic research, we consolidated the raw resequencing data of 1465 soybean genomes available in the public and 91 highly diverse wild soybean genomes newly sequenced. These altogether provided a collection of 1556 sequenced genomes of 1501 diverse accessions (1.5 K). The collection comprises of wild, landraces and elite cultivars of soybean that were grown in East Asia or major soybean cultivating areas around the world. Our extensive sequence analysis discovered 32 million single nucleotide polymorphisms (32mSNPs) and revealed a SNP density of 30 SNPs/kb and 12 non-synonymous SNPs/gene reflecting a high structural and functional genomic diversity of the new collection. Each SNP was annotated with 30 categories of structural and/or functional information. We further identified paired accessions between the 1.5 K and 20,087 (20 K) accessions in US collection as genomic “equivalent” accessions sharing the highest genomic identity for minimizing the barriers in soybean germplasm exchange between countries. We also exemplified the utility of 32mSNPs in enhancing post-genomics research through in-silico genotyping, high-resolution GWAS, discovering and/or characterizing genes and alleles/mutations, identifying germplasms containing beneficial alleles that are potentially experiencing artificial selection.

**Conclusion:**

The comprehensive analysis of publicly available large-scale genome sequencing data of diverse cultivated accessions and the newly in-house sequenced wild accessions greatly increased the soybean genome-wide variation resolution. This could facilitate a variety of genetic and molecular-level analyses in soybean. The 32mSNPs and 1.5 K accessions with their comprehensive annotation have been made available at the SoyBase and Ag Data Commons. The dataset could further serve as a versatile and expandable core resource for exploring the exponentially increasing genome sequencing data for a variety of post-genomic research.

**Supplementary Information:**

The online version contains supplementary material available at 10.1186/s12864-022-08326-w.

## Background

Soybean [*Glycine max* (L.) Merr.] is one of the most economically important field crops for its high seed protein (40%) and oil (20%), which are primarily used for animal feed, human consumption and industrial use. Soybean also plays a vital and sustainable role in agriculture through fixing atmospheric nitrogen. The worldwide soybean production has been tripled and its growing acreage has doubled since 1993 [1]. However, the demand for soybean due to its better nutritional value is predicted to continuously increase with an ever-growing world population [2]. It is critical to explore soybean genetic diversity for developing new cultivars to meet the rising demand.

With the release of the Williams 82 soybean reference genome sequence assembly in 2010 [3], a SoySNP50K iSelect Bead Chip containing 52,041 genome-wide SNPs was developed through genome resequencing of eight soybean accessions [4]. It was used to genotype 20,087 soybean accessions available in USDA soybean germplasm collection. The SoySNP50K genotyping data has become a valuable resource for a range of soybean genetics research [5–8]. In addition, transcriptome sequencing has been explored to examine the myriad transcript sequence and accumulation variations present in diverse soybean accessions. The transcriptome diversity analysis has successfully identified single nucleotide transcript variants and large DNA InDels that causes soybean seed quality and maturity variations [9–11]. With the continuous reduction in next-generation sequencing cost, a large number of soybean accessions have been re-sequenced for a wide range of soybean genome research such as developing soybean pan-genomes and understanding dynamic changes of soybean genomes [12–17]. The availability of soybean whole-genome and transcriptome sequencing data provides an unprecedented opportunity to access the genomic and genetic variation at a single nucleotide resolution in a large population and allows to explore and develop new strategies for soybean genetic improvement. Our recent analysis of 631 consolidated genome sequences from different sequencing studies facilitated uncovering of a *SWEET* gene underlying a major large-effect protein and oil QTL on chromosome 15. It supports that a two-nucleotide deletion allele in *SWEET* gene has been selected through domestication and used in breeding program to improve protein content in soybean [18]. However, most of the large-scale genome sequencing studies only focus on a specific set of accessions. Not only this, but most of the data are also released in a raw sequencing reads format in the public database like Sequence Read Archive (SRA) of the NCBI (https://www.ncbi.nlm.nih.gov/sra). However, most of laboratories lack the capacity and affordability to consolidate and systemically analyze such a huge amount of raw sequencing data available in public [19], and thus these valuable resources are relatively under-utilized. Henceforth, there is a pressing need to develop a technology platform to consolidate these massive whole-genome sequencing datasets available in the public, and systematically analyze them using the same bioinformatics pipelines and/or criteria to generate an expandable and user-friendly public-accessible resource for research community.

Soybean (*G. max*) was domesticated from *G. soja* in China about 6000 years ago [20, 21]. Artificial selection during domestication and breeding dramatically reduced the genetic diversity in modern soybean cultivars [12, 14, 22]. It has been suggested that North American soybean varieties underwent severe genetic bottlenecks [22]. Loss of genetic diversity in cultivated soybean species has been imposing a great challenge for improvement of new trait genes/variation in soybean. A recent study also revealed that genetic diversity does not increase significantly beyond approximately 800 *G. max* accessions [19]. In contrast, wild accessions possesses a high genetic diversity [12, 14, 23, 24] and is a rich genetic resource for developing improved soybean cultivars with traits like salt tolerance, cyst nematode resistance, or increased protein content [7, 18, 25, 26]. However, the number of wild soybean accessions being sequenced is very limited. To increase genome diversity for discovering novel and superior trait genes/gene variants and gain insight into soybean domestication process, it is imperative to include additional diverse wild accessions [14, 24, 27].

In this study, we analyzed the integrated publicly available genome sequences of 1465 cultivated soybean accessions and an in-house generated sequence data of 91 *G. soja* accessions representing the *G. soja* diversity in the US Soybean Germplasm Collection [12–15]. Our in-depth analysis identified and annotated 32 million SNPs (32mSNPs) across the genomes of diverse 1556 (1.5 K) soybean germplasm lines (*G. soja* accessions, landraces and improved cultivars) collected worldwide. In addition, we characterized and revealed the population structures and genetic diversity of the 1.5 K accessions. We demonstrated various utilization of the dataset for both basic and applied research in soybean. The identified variants have been made available to the research community in the public repository in a user-friendly Variant Call Format (VCF). The collection of 32mSNPs from 1.5 K accessions can serve as a valuable resource for untapping value of the huge amount of soybean genome sequencing data and exploring the genome diversity of the 1.5 K diverse soybean accessions for various soybean basic and applied research. The resource could be continuously updated with the newly generated sequencing data as additional genome sequencing data are available in the public.

## Results

### A consolidated collection of 1501 diverse soybean accessions

We retrieved approximately 15 terabytes of whole-genome sequencing reads of 1465 genomes from the NCBI SRA database. The sequencing depths ranged from 2.7- to 65.1-fold genome coverage (Table S1). Our in-house sequenced whole-genomes of 91 diverse *G. soja* accessions were at a depth ranging from 7.4 to 41.3-fold genome coverage. The 91 *G. soja* accessions represent the overall diversity of 1168 *G. soja* accessions in the US Soybean Germplasm Collection based on the genetic distances, geographic locations, and maturity groups [5]. In total, the sequencing reads of 1556 soybean genomes were generated from 1501 accessions (hereafter referred to 1.5 K).

**Fig. 1:**
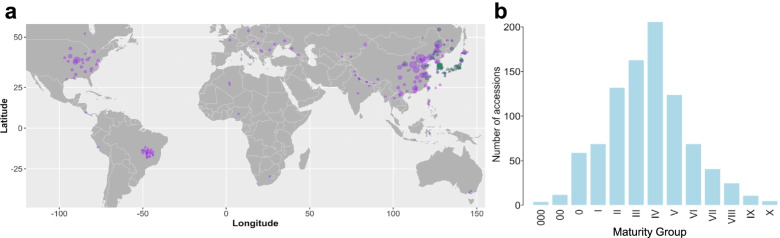
Origins and maturity groups of the 1.5 K soybean accessions. **a** Geographic distribution of the 1.5 K collection worldwide. The dots in green denote *G. soja* and those in purple denote *G. max*. Dot size denotes the sample size at the indicated geographic region. **b** Distribution of maturity group for all the accessions. Each bar represents the number of accessions belonging to the corresponding maturity group

Out of the 1.5 K accessions, a total of 1194 accessions were annotated for their germplasm types as *G. soja* accessions (204 accessions), landraces (472), and improved cultivars (518) (Table S1). The 1.5 K accessions were from a wide range of geographic locations across the world, including major soybean growing countries (Fig. 1a and Table 1). Accessions from China and the United States accounted for majority of the accessions (71.3%) with larger set from China (967 accessions) than from United States (165 accessions). The remaining 28.7% (424 accessions) accessions were collected from the other 37 countries (Table S1). This collection also included 32 accessions from Brazil, one of those largest soybean producing countries [28]. *G. soja* accessions were mainly from East Asian regions (China (71), South Korea (61), Japan (47) and Russia (25). In addition, the 1.5 K accessions were found to be distributed into 13 maturity groups (MGs 000-X) with majority (67.93%) of the collection in MGs II, III, IV, and V (Fig. 1b and Table S1). Thus, this 1.5 K collection comprising of accessions from world over harbors abundant soybean genetic diversity. Inclusion of the in-house sequenced diverse 91 *G. soja* accessions would greatly increase genetic diversity of the soybean collection and enable us to tap diversity retained in wild soybean.

**Table 1:** The origins of 1.5 K soybean collection

Origins	No. of accessions	Origins	No. of accessions
China	955	Nepal	2
United States	155	Former Serbia and Montenegro	2
Japan	105	Serbia	2
South Korea	93	Indonesia	2
Russian Federation	42	Netherlands	1
Brazil	32	Peru	1
North Korea	17	Georgia	1
Canada	14	Kyrgyzstan	1
India	8	Belgium	1
Vietnam	6	Italy	1
Moldova	4	Nigeria	1
Ukraine	3	Myanmar	1
Germany	3	South Africa	1
Romania	3	Uzbekistan	1
Algeria	2	Sweden	1
France	2	Costa Rica	1
Philippines	2	Austria	1
Australia	2	Poland	1
Thailand	2	Unknown	27
Hungary	2	Total	1501

Duplicated-sequenced genomes were counted once here

### Identification and annotation of 32mSNPs among 1.5 K diverse soybean accessions

**Fig. 2:**
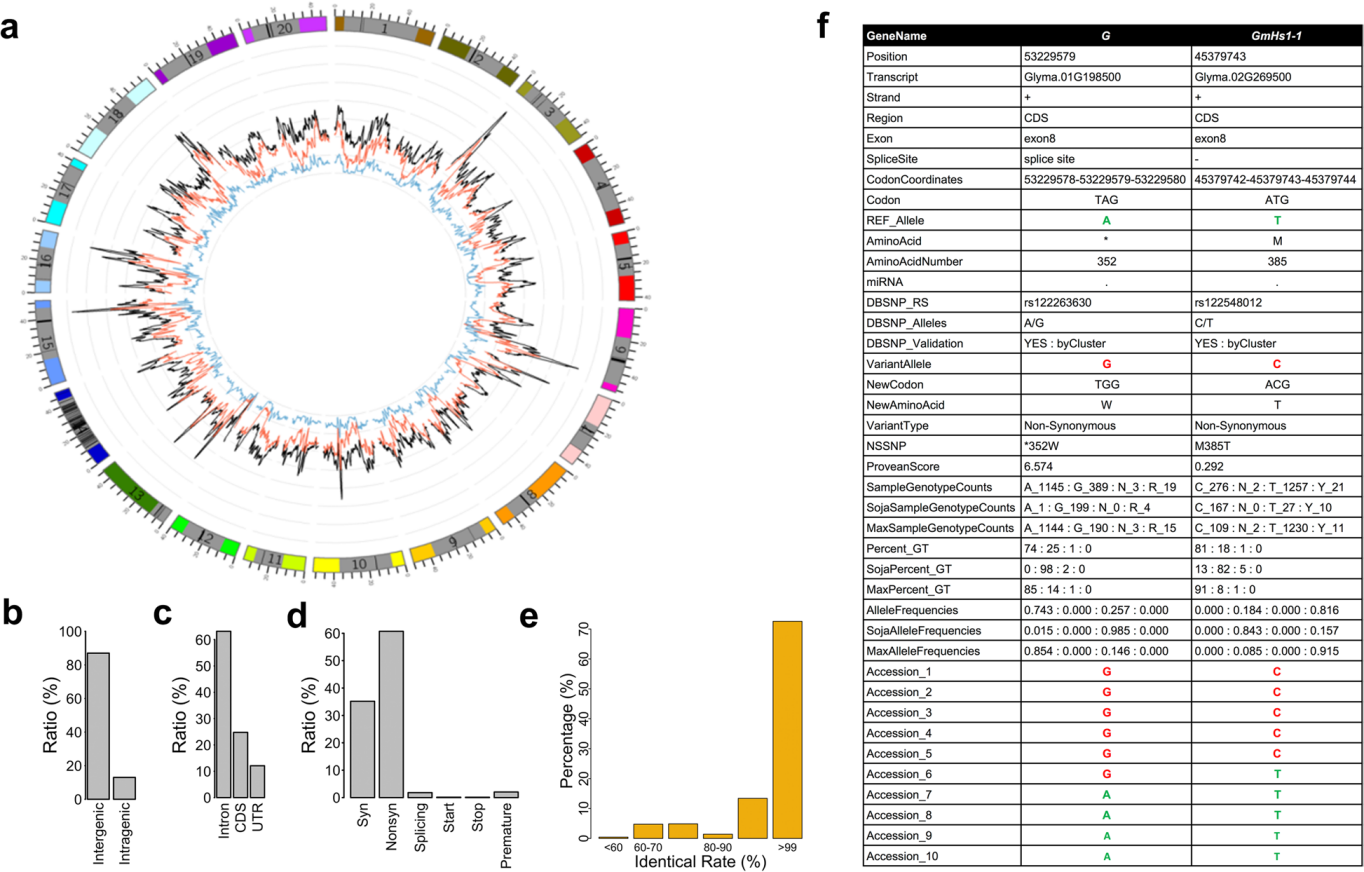
Annotation of the 32mSNPs. **a** Distribution of the SNPs along 20 chromosomes compared with the dbSNPs. The outermost circle represents the 20 soybean chromosomes. 0, 20, and 40 outside the circle represent 0 Mb, 20 Mb, and 40 Mb positions on the chromosome, respectively. The solid gray boxes and black bars indicate pericentromeric regions and centromeric repeats, respectively. The black, red, and blue curves of the inner circle showed the distribution of the SNP set identified in the 1.5 K accessions, soybean dbSNPs, and the subset of SNPs with minor allele frequency ≥ 0.25, respectively. **b** Percent of the SNPs in the 32mSNPs in genome features (intergenic and intragenic). **c** Percent of genic SNPs in the 32mSNPs (Intron, CDS, UTR). **d** Percent of synonymous (Syn), non-synonymous (Nonsyn) SNPs, and the SNPs in splicing sites, start codons, stop codons, and the SNPs causing premature in the 32mSNPs. **e** Percent of identity at the coordinate-matched SNPs for the accessions that have been genotyped by genome-resequencing and SoySNP50K. **f** A SNP report exemplified with two previously identified domestication genes. *G* encodes a stay-green gene controlling soybean dormancy and *GmHs1-1* encodes calcineurin-like protein controlling hard-seededness. The SNP report contains 30 annotation categories for each identified SNP in the 32mSNPs. Reference and alternative alleles were highlighted in green and red, respectively

We analyzed a total of 208.3 billion paired-end sequencing reads of the 1556 genomes with an average of 133 million reads and 14-fold genome coverage per accession (Table S1). A total of 32,456,244 SNPs (designated as 32mSNPs) were discovered at an average SNP density of 30 SNPs/kb. Of these, 16.3 million and 8.4 million SNPs had minor allele frequency (MAF) higher than 0.01 and 0.05, respectively. We revealed a significantly higher density of SNPs in euchromatic regions than heterochromatic regions (Fig. 2a). Approximately, 87% of the 32mSNPs located in the intergenic regions. The rest 13% (5,193,083) were in the genic regions with an average density of 93 SNPs per gene (Fig. 2b). Of the genic SNPs, 12.1% and 63.1% located in untranslated regions (5′ and 3’UTR) and introns, respectively (Fig. 2c). The remaining 24.8% of the genic SNPs were present in the coding sequences (CDS) (Fig. 2c). We observed that 63% (657,371) of these SNPs in the CDS were non-synonymous. PROVEN algorithm predicted 10.7% (70,143) of the non-synonymous SNPs as deleterious with PROVEN score ≤ − 4.1 that have high probability of altering gene functions [29]. Additionally, we discovered 19,506 SNPs at splicing sites, 1562 at start codons, 1465 at stop codons, and 22,076 producing premature stop codons (Fig. 2d). Importantly, 99% of the 56,044 gene models in the soybean genome (*Wm82.a2 v1*) carried at least one non-synonymous SNP with an average of 12 non-synonymous SNPs/per gene. The collection of 1.5 K accessions are highly diverse, and likely contained at least one nucleotide mutation/allele for each gene that potentially alter gene functions and cause phenotypic variation. The collection of accessions also enable us to explore and discover the novel causative gene/ allelic variants for agronomically important traits for soybean improvement.

For the 56 accessions that were sequenced twice in different studies, the duplicated sequences for the same accessions had an average of 99.5% of sequence identity, indicating high reproducibility and accuracy of the SNP calling method utilized in the present study. Out of the 1.5 K accessions, 926 accessions have also been genotyped using Soy50KSNP Chip in a separate study [5]. We observed that 73% of 926 accessions had > 99% identity at SNP positions genotyped by both platforms (Fig. 2e). The remaining 27% of the accessions showed less than 99% identity between the two platforms. Having compared the 32mSNPs with 15,623,492 registered soybean SNPs available in the NCBI dbSNP database (https://www.ncbi.nlm.nih.gov/snp/), we observed that 13 million SNPs were present in the dbSNP database. Thus, 19 million out of the 32mSNPs represented novel SNPs.

To provide a comprehensive description of each SNP, especially for those non-synonymous SNPs potentially important to gene functions, each SNP was annotated with 30 categories of structural and functional information such as the SNP position, reference and alternative alleles, and their allelic frequency in *G. soja* and *G. max*. (Fig. 2f). *G* and *GmHs1-1*, are two agronomically important genes [30, 31] (Fig. 2f). *GmHs1-1* encodes a calcineurin-like protein controlling hard-seededness and *G* gene has been associated with dormancy in soybean. The causative reference and alternative alleles for *GmHs1-1* (reference T/ alternative C) and *G* (A/G) were identified as homozygous alleles in an average of 98.6% of 1.5 K collection. The annotation of each of these alleles in two genes between two subpopulations of *G. max* and *G. soja* revealed their highly biased distribution among the subpopulations. For example, the *G* gene had 85% of A allele in *G. max* and 98% of G in *G. soja*. This result is consistent with the previous reports showing that both genes and their alleles are associated with soybean domestication. The comprehensive annotation is therefore a highly valuable step for the post-genomic research including population-scale characterization of genome-wide SNPs and genes for discovering domestication-associated genomic loci of interest. These results also suggest the high coverage and robustness of the 32mSNPs.

### Population structure of 1.5 K soybean accessions

**Fig. 3:**
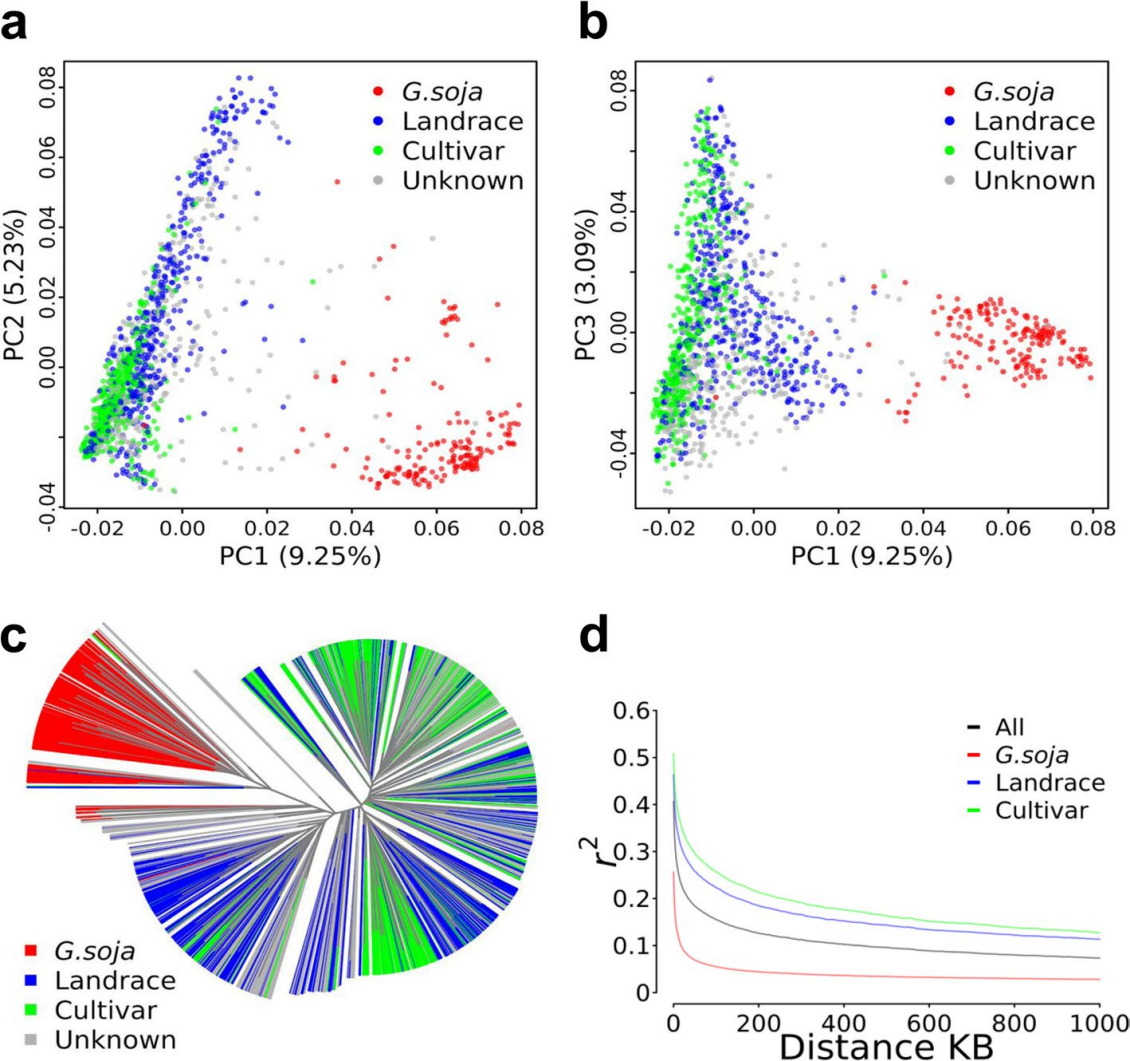
Population genomic analysis of the 1.5 K collection. **a** Scatter plots for the first two components (PC1, PC2) of the 1556 (1.5 K) accessions, and **b** for PC1 and PC3. **c** A neighbor-joining phylogenetic tree of the 1.5 K accessions. The germplasm types (*G. soja*, landrace, cultivar) are correspondingly labeled. **d** Comparison of LD decay among three germplasm types (*G. soja*, landrace, cultivar)

We assessed the population structure using principal component analysis (PCA) and neighbor-joining (NJ) phylogenetic analysis. The PCA revealed that the first 20 principal components (PCs) of the genetic data captured 32.53% of the total variance among the 1.5 K accessions. The first PC captured 9.25% of the variation and mainly explained the divergence between *G. max* and *G. soja* (Fig. 3a). The second PC captured 5.23% of the variation, mainly explaining variation within the germplasms, and the third PC captured 3.09% variation and mainly explained the variation within the remaining variation within the germplasms (Fig. 3b). The neighbor-joining phylogenetic tree constructed with the 1.5 K accessions showed general consistency of population structure corresponding with PCA, where accessions were clustered based on the germplasm types with some percentage of admixture between germplasms (Fig. 3a, b, c). *G. soja* accessions were mainly clustered independent of *G. max* clusters. As expected, we observed more diverse between *G. soja* and *G. max* groups than between landrace and cultivar within *G. max* group. Therefore, this collection has a clear, germplasm type-inferred population structure that allows to leverage the high-resolution SNPs to investigate complex processes of soybean domestication and improvement.

The estimation of linkage disequilibrium (LD) across the three germplasm types (Fig. 3d) revealed an overall rapid LD decay in *G. soja* compared to domesticated *G. max* landraces and cultivars analyzed independently. Overall LD decay of all accessions (all three germplasm types) was ~ 35 kb at *r*^*2*^ of 0.2. The LD decayed in *G. soja* at ~ 2 kb at *r*^*2*^ of 0.2, which is dramatically shorter than ~ 151 kb for landraces and much shorter than ~ 255 kb for cultivars (Fig. 3d). This result indicates that the highly diverse *G. soja* accessions in the collection greatly reduced the sizes of LD blocks, thereby, it should significantly increase the resolution of association mapping by breaking long LD in *G. max* population alone.

**Fig. 4:**
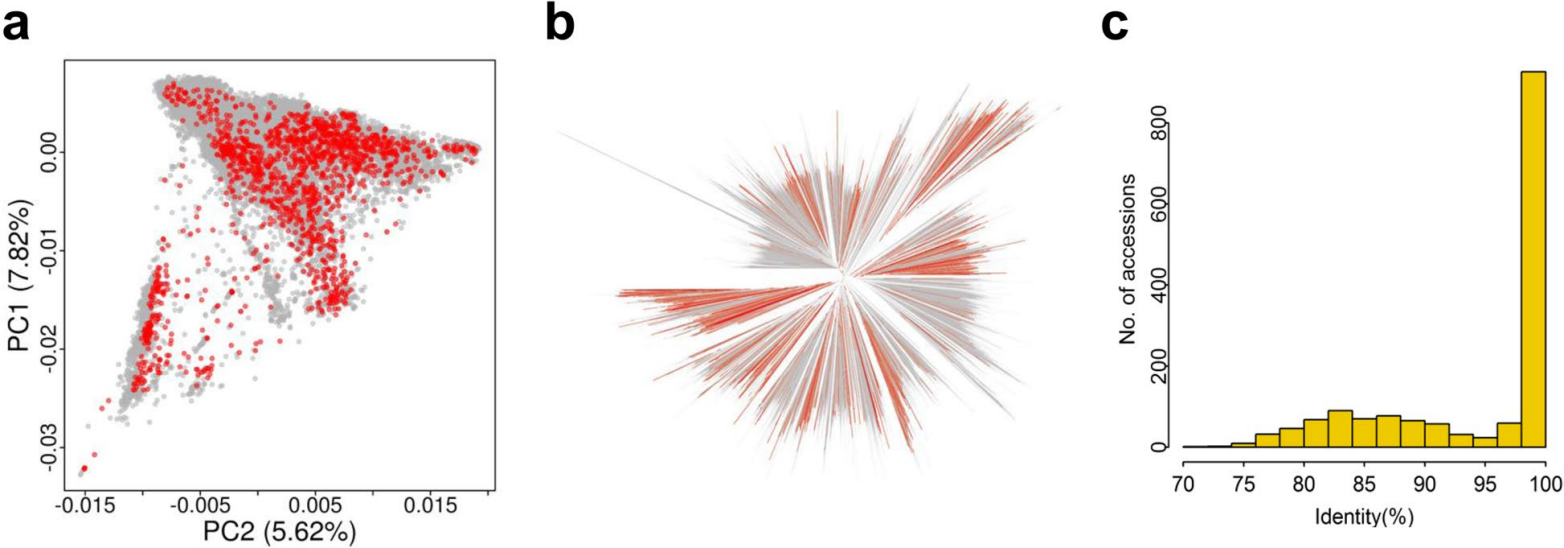
Representation of the 1556 accessions in the US 20 K Soybean Collection. **a** Distribution of the whole-genome sequenced 1556 (1.5 K) accessions and the 20,087 (20 K) accessions using principal component analysis. Red dots denote the 1.5 K accessions and gray dots denote the 20 K accessions. **b** A neighbor-joining phylogenetic tree constructed by the 1.5 K accessions and 20 K accessions. Branches in orange denote the 1.5 K whole genomes sequenced accessions and those in gray denote the 20 K accessions. **c** The genomic similarity between the 1.5 K accessions corresponding to the 20 K accessions as determined by comparing the identity of coordinated-matched SNP sites

To determine if the 1.5 K diverse collection can reflect genetic diversity of the 20 K wild and cultivated accessions in the US Soybean Germplasm Collection, which have been genotyped by SoySNP50K Chip. PCA analysis showed that the 1.5 K accessions spread in major clusters among the 20 K accessions (Fig. 4a). This observation was supported by the presence of the 1.5 K accessions (orange) in almost all major clusters in the neighbor-joining phylogenetic tree of the 20 K accessions (Fig. 4b). Having conducted a pairwise comparison between the two collections of accessions, we identified the accessions in the 1.5 K collection that shared the highest sequence identity with each of the 20 K accessions (Fig. 4c, Table S1). These accessions were treated as potentially genomic “equivalent” accessions in US collection. These can therefore serve as a reference for an inaccessible sequenced accession or may be the genome sequence of the un-sequenced accessions to maximize the use of the genome sequences generated worldwide for soybean research (Table S2).

### Diverse application of the 32mSNPs among 1.5 K soybean genomes

The 32mSNPs among 1.5 K diverse wild and cultivated soybean accessions offers an excellent resource for a variety of post-genomic research. Here we demonstrated that it can be effectively used for high-resolution association analysis and reverse genetic study.

#### High-resolution association analysis

**Fig. 5:**
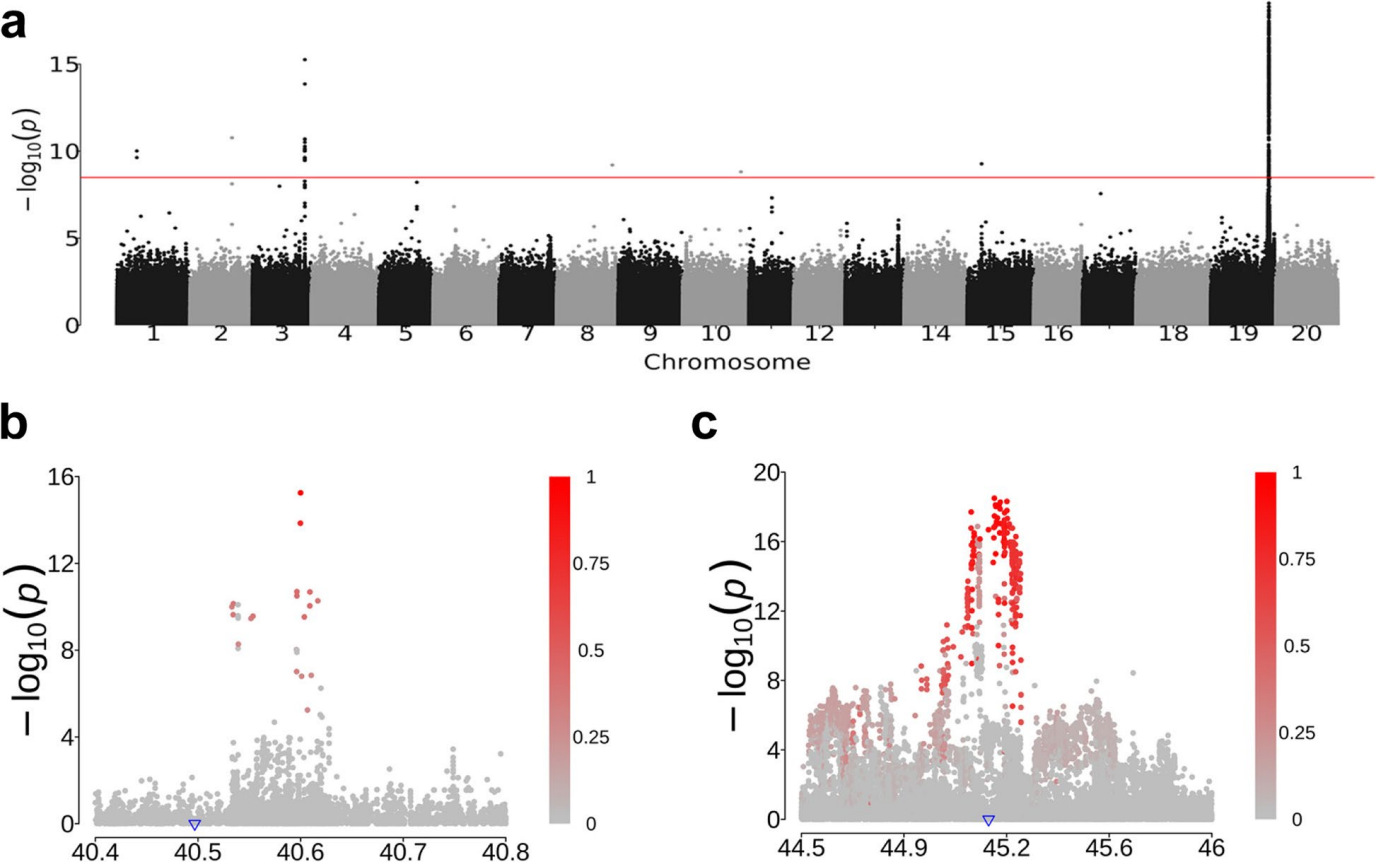
Two associations for stem determinate using the 32mSNPs. **a** A Manhattan plot illustrating two major QTLs associated with stem determinate on chromosomes 3 and 19. **b** and **c** Zoomed-in Manhattan plot for the association regions on chr3 and chr19, respectively. Blue triangles denote the physical positions of the *Dt1* and its homologous gene. The color intensity of each SNP indicates its *r*^2^ value with the peak association SNP. The color scale was shown beside the panel

SNPs have been one of the most widely used molecular markers in genetic research. The 32mSNPs among 1.5 K diverse soybean accessions will not only be advantageous in high-resolution SNPs-based large and diverse population diversity study but also in the QTL discovery studies for traits of interest using genome-wide association study (GWAS). The determine growth was phenotyped in 642 accessions out of the 1.5 K accessions. GWAS with the 642 accessions revealed two major QTLs with strong associations (*p* < 7.10 × e^8^) on chromosomes 3 and 19 (Fig. 5a). A cluster of SNPs spanning 200 kb (chr19: 45105190-45305190) were significantly associated with determinate growth at the QTL on chr19. These significantly associated SNPs were found to be co-located with a previously reported *Dt1* gene (*Glyma.19G194300*) underlying a stem determination QTL [32, 33] (Fig. 5c). Interestingly, a cluster of strongly associated SNPs at the QTL region on chr 3 nearly coincided with a *Dt1* paralog (*Glyma.03G194700*) (Fig. 5b). The GWAS result strongly supports that the *Dt1* paralog is likely to preserve its function in regulating the determination growth after gene duplication [32]. The successful pinpointing of the determinate growth QTLs to a previously known *Dt1* gene and its paralog using 32mSNPs genotyping data among 1.5 K accessions indicates that these dataset are quite effective for performing high-resolution GWAS of a trait of interest to discover their causative QTL genes and alleles.

#### Reverse genetics to explore variation of trait genes

Nucleotide substitution is one of the major DNA variants leading to gene function alteration and plant phenotypic variation. The 32mSNPs among 1.5 K diverse soybean accessions with comprehensive structural and functional annotation offers an excellent resource to study gene functions and identify causative alleles for a trait gene using a reverse genetic approach. It also allows us to develop and apply a facile in-silico approach to genotype given genes/features or defined genomic regions of interest for 1.5 K soybean accessions. For example, *FATTY ACID DESATURASE 2* (FAD2) is a key enzyme in fatty acid biosynthetic pathways and plays an important role in regulating fatty acid profile in soybean seeds. This FAD2 enzymes are encoded by seven *FAD2* genes in soybean [34]. We identified a total of 340 SNPs in *FAD2* genes. Out of the 340 SNPs, 53 non-synonymous SNPs, 2 nonsense SNPs, and 2 SNPs at splicing sites were identified (Table 2). Four SNPs (S86F, M126V, P137R, and I143T) were previously demonstrated to be highly correlated with high oleic acid content [35, 36]. We identified three (S86F, M126V, P137R) of the four SNPs, indicating a high diversity and coverage of the SNPs among 1.5 K collection. Of all these variants, the missense mutation, P137R in *FAD2-1B* in PI283327 (Pingtung Pearl, maturity group V) is a critical allele used for breeding high oleic acid [35]. Remarkably, despite rare alleles, we also successfully identified another accession (PI506933, Kouiku at maturity group IV) carrying the same P137R allele (Table 3). This result suggests the importance of keeping all identified SNPs, including rare alleles among 1.5 K collection for further studies. These altogether demonstrated that the collection of highly diverse 1.5 K soybean accessions along with in-silico genotyping offers a highly effective approach for discovering novel or known germplasm lines containing previously identified or new trait gene mutations/alleles.

**Table 2:** Total SNPs identified in *FAD2* family genes using the 32mSNPs

	Total	FAD2-1A	FAD2-1B	FAD2-2A	FAD2-2B	FAD2-2C	FAD2-2D	FAD2-2E
Total Genic SNPs	340	16	60	22	23	144	67	8
CDS^a^	91	7	19	19	9	20	8	9
Non-Synonymous	53	6	8	13	3	11	5	7
Synonymous	38	1	11	6	6	9	3	2
Splice Site	2	1	0	1	0	0	0	0
Premature Termination Codon	2	0	0	1	0	1	0	0
Five Prime UTR^b^	55	5	25	0	10	10	5	0
Three Prime UTR	11	1	3	0	3	4	0	0
Intron	191	3	13	4	1	116	14	0

^a^Coding sequence

^b^Untranslated region

**Table 3:** Accessions carrying the DNA variant (reference allele C to alternative allele G) in *FAD2-1B* that results in amino acid change at P137R

PI	Common Name	Species	MG	P137R
PI518671	Williams82	*G. max*	III	C
PI283327	Pingtung Pearl	*G. max*	V	G
PI506933	Kouiku 1	*G. max*	IV	G

## Discussion

The economic importance of soybean in agriculture and food industry has promoted the whole-genome sequencing of thousands of cultivated and wild soybean accessions globally. To facilitate applying the huge amount of sequencing data into soybean research and improvement, this study consolidated the large quantity of genome sequencing data generated in independent studies into one dataset, and made them publicly available in a user-friendly format and demonstrated their versatile applications [37]. It has been long acknowledged that soybean has a vast amount of genetic diversity [31]. The increased size and diversity of the consolidated population should greatly enhance statistic power for post-genomic population studies such as GWAS to identify trait genes and causative alleles. However, analysis of such massive genome sequences requires an investment of tremendous time and effort, the proficient bioinformatics skills, and high-performance computing. These have been limiting the use of the valuable genome sequencing data in post-genomic research. Identification and comprehensive annotation of the32 million SNPs among the 1.5 K soybean accessions using the same bioinformatic pipelines and parameters will greatly enhance the translational genomic research.

The collection of 1.5 K accessions include many soybean germplasm lines widely used in soybean genetic studies and breeding programs such as parental lines of Nested Association Mapping and many RIL mapping populations, important landrace used in the soybean breeding, and elite cultivars [18, 38–41]. For example, Lee (PI548656), Essex (PI548667) and Harosoy (PI548573) that have served as important breeding materials in Southern and Northern US breeding programs are part of the collection. Thus, the collection of accessions should contain genetic diversity important to current soybean breeding and genetic studies. Incorporation of 91 representative wild accessions in the collection can enriched the gene pools for new allele discovery and evolutionary research [12, 17, 18]. The genotyping data at 32mSNP positions among 1500 diverse soybean accessions with an average density of 30 SNPs/Kb genome sequence enable detailed genomic analyses at a single nucleotide resolution. We observed an average of 12 non-synonymous SNPs per gene indicating that the 1.5 K accessions is a rich resource of gene mutants and can be useful for gene function discovery.

**Fig. 6:**
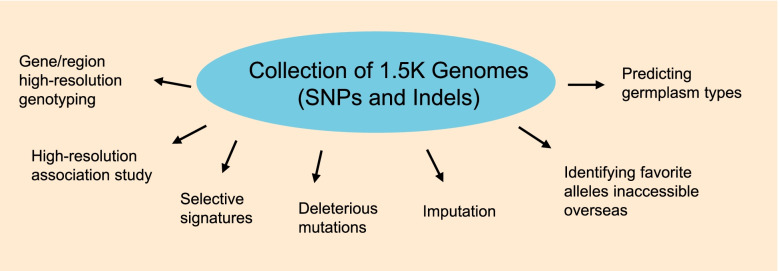
Versatile Usages of the 32mSNPs in the collection of 1.5 K genomes

The usage of the 32mSNPs genotyping data in the 1.5 K soybean germplasm population may beyond what is presented here (Fig. 6). For example, the 1.5 K covers a significant amount of genetic diversity in the US collection of 20 K accessions. Therefore, imputing the Soy50KSNP chip data based on 20 K accessions with the 32mSNPs among 1.5 K accessions may enable in-depth exploration of the entire USDA Soybean Collection [42]. The 32mSNPs may also contribute to further analysis of genome patterns such as structural variation [43], selective sweeps, and deleterious mutations in soybean [14, 16, 17] or comparative studies with a wider breadth of species. Combining our detailed analysis with other studies such as haplotype map-based GmHapMap [19] and other types of variation such as structural variation in soybean accessions [16] could be more powerful to address a broad range of basic and applied research questions. To leverage full potential of the SNP data among 1.5 K accessions, the dataset with its detailed annotation information was made public at SoyBase (https://soybase.org) and Ag Data Commons (10.15482/USDA.ADC/1519167) for extensive uses. Identification of genomic “equivalent”/closely related accessions between 1.5 K and 20 K accessions of US soybean collection offers an opportunity to maximize the use of genomic data, and allowsto access useful variation existing in the accessions present overseas and are inaccessible due to international germplasm exchange policy barriers. In addition, the phylogenetic distances as indicated in the phylogenetic tree may provide guidance in appropriate selection of representative parents to develop population for genetic studies or breeding [44]. Despite an estimated average 2.63% false positive/negative rate [12] and the dramatic drop in the number of high-quality SNPs after MAF-based filtration (0.01 and 0.05), it is advisable to retaining rare alleles in the collection. This is useful to identify not only rare but valuable alleles or accessions as exampled by the identification of two P137R-carrying accessions (MAF = 0.12%). Therefore, the 32 million SNPs among the 1.5 K diverse soybean accessions may be an indispensable rich source for mining valuable trait genes and their variants. Recently, additional genome sequences were released for examining the pan-genome and domestication-associated variation [16, 17, 45]. With the genome sequencing analysis pipeline developed in the study, it could serve a core resource for continuous integration of those genome sequencing data newly available in the public to maximize application of the huge amount of genome sequencing data into soybean research and product development.

In summary, this study provides a user-friendly resource of soybean genomic variants at a single nucleotide-resolution and examples for its versatile usages such as trait and gene variation discovery through genetic association studies, thereby aiding soybean fundamental research and crop improvement (Fig. 6).

## Materials and methods

### Data collection and genome sequencing

Genome sequencing reads of all *G. soja* and *G. max* accessions that were generated and made available in previous studies until 2020 [12–15] were retrieved from the Short Read Archive (SRA) database at NCBI (www.ncbi.nlm.nih.gov) using the fastq-dump sra toolkit (https://github.com/ncbi/sra-tools). The sequencing reads of 91 representative *G. soja* accessions (Table S1) [4] were generated using the Illumina HiSeq2000 sequencer. All sequencing data were combined for further analysis. Based on PI (Plant Introduction) information, we identified that sequencing of 56 accessions were performed twice and one was sequenced thrice from different laboratories (Table S1). Considering the possible genetic variation in the duplicated accessions from different laboratories, we retained all the multiple sequences of these accessions in our analysis but accounted different sequences corresponding to one of these accession as individual accessions. The information on accession ID or name, species, maturity group, origin, and geographical origin was retrieved from the Germplasm Resources Information Network (GRIN, https://www.ars-grin.gov/) and listed in Table S1. The Williams 82 soybean reference genome sequence was downloaded from the Phytozome v12 (https://phytozome.jgi.doe.gov/pz/portal.html). The NCBI’s dbSNP Data were downloaded at https://www.ncbi.nlm.nih.gov/SNP. The SoySNP50K iSelect Bead Chip for 20,087 (20 K) soybean accessions in the US Soybean Collection were downloaded from the SoyBase (https://soybase.org).

### SNP calling and annotation

The raw sequencing reads were aligned with soybean reference genome (*G. max* cv. Williams 82.a2 v1) available at Phytozome v12 [3, 46] using burrows-wheeler aligner (BWA) (version: 0.7.17-r1188) [47]. Picard tool was used to add reads group, reorder and sort the reads, and mark the duplicated ones (version: 2.9.2, https://broadinstitute.github.io/picard/). SNPs were called using UnifiedGenotyper function of Genome Analysis Toolkit (GATK) (Version: 3.4) with default parameters [48]. SNPs in each sample were filtered as follows: a minimal coverage of 20%, read depth ≥ 5 reads, SNPs quality score of at least 50 and, maximum of 2 SNPs across 10-bp window. Each sample was genotyped individually at each of the SNP sites passing the filtering criteria. SNP sites for the 1.5 K collection were merged and further filtered based on minor allele frequency > 0.001 and missing rate < 0.5. We used the relaxed filtering criteria to retain rare alleles for downstream research. The genome-wide distribution of these high quality SNPs was illustrated using Circos [49]. A custom Perl script was developed to annotate SNPs based on the coordinates of features (intron, exon, UTR, splicing site, *etc*) of all 56,044 gene models from *G. max* cv. Wm82.a2.v1 reference genome [3, 46]. The effects of non-synonymous SNPs on gene functions were predicted by Protein Variation Effect Analyzer (PROVEAN version: 1.1.5) [29].

### SNP data comparison

SoySNP50K iSelect Bead Chip based genotyping results for 20,087 (20 K) accessions of the US Soybean Collection were downloaded from the SoyBase (https://soybase.org) [4]. Using custom Perl scripts, the SNP coordinates obtained from both SoySNP50K iSelect Bead Chip and our 32mSNPs were identified and used to compare the identity rate of accessions genotyped by these two technology platforms. Coordinates with either no call or N were not included in the comparison. The percent identity of SNP coordinates among accessions was calculated based on the ratio of Sum of Identical SNPs and All Coordinates Compared Count.

The 15,623,492 registered soybean SNP coordinates were downloaded from the NCBI’s dbSNP Database (https://www.ncbi.nlm.nih.gov/SNP) and compared with the 32mSNPs from 1.5 K genomes. The coordinates of 32mSNPs that did not correspond with those in dbSNP database were regarded as newly identified SNPs.

### PCA analysis

Principal component analysis (PCA) was used to analyze population structure and relation between re-sequenced 1556 accessions and the 20,087 accessions in the GRIN database using filtered 32mSNPs and SoySNP50K SNPs, respectively. PCA was conducted using the SNPRelate package [50]. An identity-by-state (IBS)-based neighbor-joining (NJ) phylogenetic tree was constructed for all accessions using an R package SNPRelate [50]. The phylogenetic tree was visualized using *ape* packages [51]. All SNPs used in this analysis were filtered using *snpdsLDpruning* function of SNPRelate based on ld. Threshold ≥0.5, missing rate ≤ 0.2. LD decay was analyzed using PopLDdecay with -MaxDist of 1000 and MAF ≥0.05 [52].

### Genome-wide association study

The phenotypic data for stem determinate was downloaded from the GRIN database. SNP data were filtered to keep only biallelic SNP and minor allele frequency ≥ 0.01. GWAS was performed using a linear mixed model in GAPIT [53, 54] with Model.selection = T. Kinship was calculated using the default setting in GAPIT and included in the analysis. The threshold for significant association was determined with *p* < 0.05/ total amount of SNPs used in the analysis. The regional LD heatmap was generated using the LDheatmap package [55].

## Supplementary Information

**Additional file 1: Table S1.** Summary for the collection of 1.5 K accessions: For each accession, the columns showed its common name, PI, maturity group (MG), origin and geographic location (latitude and longitude), coverage of its genome sequencing, percent aligned to the reference genome sequence, total number of reads (Total), percentage of identity in comparison with its own 50 K SNP genotype, the PI with the highest Percentage of Identity (PID) in comparison with 50 K SNP genotypes of all accessions including itself and not including itself. **Table S2.** Best match of each of the 20 K accessions to the 1.5 K accessions: The best match accession and the percentage of identity is listed for each accession in US collection of 20 K accession.

## Data Availability

The dataset supporting the collusions of this article is available in the SoyBase (https://soybase.org) and Ag Data Commons (10.15482/USDA.ADC/1519167).
